# The Role of Long Non-Coding RNA NNT-AS1 in Neoplastic Disease

**DOI:** 10.3390/cancers12113086

**Published:** 2020-10-23

**Authors:** Cong Zhou, Shiwei Duan

**Affiliations:** Medical Genetics Center, School of Medicine, Ningbo University, Ningbo 315211, Zhejiang, China; congzhou@sjtu.edu.cn

**Keywords:** lncRNA, NNT-AS1, cancer, miRNA, sponge

## Abstract

**Simple Summary:**

Nicotinamide nucleotide transhydrogenase-antisense 1 (NNT-AS1), which is a newly-discovered long non-coding RNA (lncRNA), has been found to be dysregulated in a variety of neoplastic diseases. With the accumulation of studies on NNT-AS1 in recent years, the mechanism of NNT-AS1 and its significance for tumor occurrence and progression are constantly being updated and improved. Thus, this paper aims to summarize the abnormal expression of NNT-AS1 and its prognostic values in different neoplastic diseases. In addition, the detailed competing endogenous RNA networks and subsequent biology behaviors, as well as the role of NNT-AS1 in mediating cisplatin resistance are revealed in this paper. This review not only summarizes the past research of NNT-AS1, but also provides some ideas for future research in this field.

**Abstract:**

Studies have shown that non-coding RNAs (ncRNAs), especially long non-coding RNAs (lncRNAs), play an important regulatory role in the occurrence and development of human cancer. Nicotinamide nucleotide transhydrogenase-antisense 1 (NNT-AS1) is a newly-discovered cytoplasmic lncRNA. Many studies have shown that it has abnormally-high expression levels in malignant tumors, but there are also a few studies that have reported low expression levels of NNT-AS1 in gastric cancer, breast cancer, and ovarian cancer. At present, the regulatory mechanism of NNT-AS1 as a miRNA sponge, which may be an important reason affecting tumor cell proliferation, invasion, metastasis, and apoptosis is being studied in-depth. In addition, NNT-AS1 has been found to be related to cisplatin resistance. In this review, we summarize the abnormal expression of NNT-AS1 in a variety of neoplastic diseases and its diagnostic and prognostic value, and we explain the mechanism by which NNT-AS1 regulates cancer progression by competing with miRNAs. In addition, we also reveal the correlation between NNT-AS1 and cisplatin resistance and the potential clinical applications of NNT-AS1.

## 1. Introduction

The Encyclopedia of DNA Elements (ENCODE) project has shown that at least 76% of the human genome is transcribed, but that only ~1.2% of RNAs are protein-coding, while the remainder have no obvious protein-coding potential (referred to as non-coding RNAs, ncRNAs) [1,2]. Initially, ncRNAs were considered transcriptional “noises”, but with the deepening of molecular biology research, more and more studies have clarified the important role of ncRNAs in various biological activities [3,4] In addition, mutations and disorders of ncRNAs, especially long non-coding RNAs (lncRNAs), have been found to be closely related to cancer [5,6]. LncRNAs were initially considered to be transcripts longer than 200 nucleotides without a functional open reading frame (ORF >100 aa) [7]. However, many recent studies have found that some transcripts previously annotated as lncRNAs contain small ORFs and can be translated into small peptides or micro-peptides, some of which have a biological function [8,9,10]. According to LNCipedia 5 (a public database for lncRNA sequence and annotation), current numbers of annotated lncRNAs have reached 127,802 [11]. In the past few decades, a large number of studies have shown that lncRNAs play an important role in the progression of various cancers and drug resistance to chemotherapy [12,13]. Therefore, abnormally-expressed lncRNA is expected to become a novel biomarker or a new target for cancer treatment.

**Figure 1:**
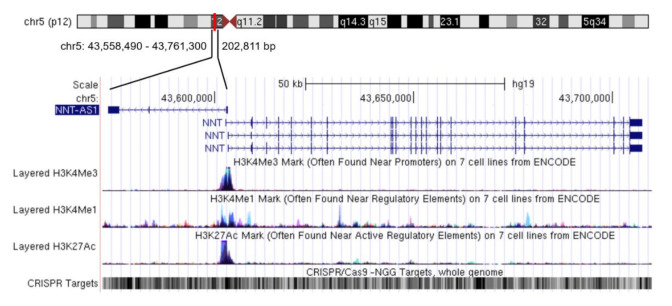
Genome position of long non-coding RNA (lncRNA) nicotinamide nucleotide transhydrogenase-antisense 1 (NNT-AS1). NNT-AS1 is mapped to chromosome 5. Reference information comes from the University of California Santa Cruz (UCSC) Genome Browser (http://genome.ucsc.edu/).

Nicotinamide nucleotide transhydrogenase-antisense 1 (NNT-AS1) is a newly-discovered lncRNA mainly distributed in the cytoplasm [14]. NNT-AS1 is located in 5p12 and has three exons. The transcription directions of NNT-AS1 and the adjacent protein-coding gene nicotinamide nucleotide transhydrogenase (NNT) are opposite and there is no overlapping region between NNT and NNT-AS1 (Figure 1). In a study of multiple sclerosis, there was no significant difference in NNT-AS1 levels in peripheral blood samples between the case group and the healthy group [15]. However, in all the studies on tumorous diseases that have been published so far, the expression level of NNT-AS1 in tumor tissues or tumor cells has been abnormal, which indicates the potential correlation between NNT-AS1 dysregulation and tumorous diseases. This review summarizes the abnormal expression levels of NNT-AS1 in tumors and describes its regulation of a large number of downstream genes by competing with miRNAs to affect multiple signaling pathways. We also summarize the diagnostic and prognostic values of NNT-AS1 in cancer and present its role in the resistance of antitumor drugs.

## 2. The Aberrant Expression of NNT-AS1 in Cancer

Current research shows that NNT-AS1 is highly expressed in most tumors, including non-small-cell lung cancer (NSCLC) [16,17,18,19], lung squamous cell carcinoma (LUSC) [20], colorectal cancer (CRC) [14], hepatocellular carcinoma (HCC) [21], cholangiocarcinoma (CAA) [22,23,24], cervical cancer [25,26], osteosarcoma [27,28], bladder cancer [29,30], and glioma [31]. These results have been verified in the corresponding tumor tissues or tumor cells. In subsequent functional experiments on cells, these studies further revealed the cancer-promoting effects of high levels of NNT-AS1, including induction of proliferation, promotion of invasion and metastasis, and inhibition of apoptosis. In addition, some studies have used mouse models to confirm the tumor growth and liver metastasis effects of NNT-AS1 (Table 1).

However, studies on the expression of NNT-AS1 in gastric, breast, and ovarian cancers seem to be contradictory. In gastric cancer, there have been three studies that have reported high levels of NNT-AS1 in tumor tissues and cell samples, and the performance of NNT-AS1 in subsequent in vivo and in vitro tests also verified its cancer-promoting function [32,33,35]. However, Farbod Esfandi et al. found that compared with the adjacent samples, the NNT-AS1 levels detected in gastric cancer samples were overall down-regulated [34]. Their further stratified analysis showed that compared with tumors without lymphatic/vascular invasion, tumors with lymphatic/vascular invasion had higher levels of NNT-AS1, which seems to be consistent with the malignant manifestations of NNT-AS1 [34]. The authors attributed these contradictions to ethnic differences [34]. However, it is worth noting that the sample size of this study was relatively small and lacked follow-up in vivo and in vitro experiments. Therefore, more samples and a more complete experimental system are needed for further verification. For breast cancer, Li et al. first reported the high expression of NNT-AS1 and confirmed it as a motivator for breast cancer progression in functional experiments [36]. In contrast, Saleh Gargari S. et al. reported the low expression of NNT-AS1 in breast cancer samples, and in subgroup analysis, the expression of NNT-AS1 in estrogen receptor (ER)-negative tumor samples was higher than ER-positive tumor samples [37]. This suggests that the expression of NNT-AS1 may be related to sex hormone receptors. A recent bioinformatics study based on Gene Expression Omnibus (GEO) data analyzed the expression of lncRNA in triple-negative breast cancer (including ER-negative), in which NNT-AS1 was also found to be down-regulated [38]. In addition, in a study on ovarian cancer, the low level of NNT-AS1 was confirmed as well [39].

Interestingly, it has been reported that, unlike Erα-receptor-negative ovarian cancer cells, treatment of Erα-receptor-positive ovarian cancer cells with estrogen (E2) will cause a large number of lncRNA disorders and changes in some protein levels, such as elevated TC0101441, which was found to promote the invasion and metastasis of ovarian cancer cells [40]. This suggests that sex hormones and sex hormone receptors may affect lncRNA and tumor progression. In Erα-receptor-positive breast cancer cells, when E2 is monitored or E2 is given, the levels of miR-424 and E2F1 also increase [41,42]. In a gastric cancer study, the regulatory axis of NNT-AS1/miR-424/E2F1 was confirmed [32]. Therefore, the low expression of NNT-AS1 up-regulates miR-424 and E2F1, and the abnormal E2 level is likely to be correlated. Although the current evidence is not enough to determine the causal relationship, this may be one of the reasons for the low expression of NNT-AS1 and the progression of sex-hormone-related tumors. In addition, in a study of papillary thyroid carcinoma, lncRNA-H19 was found to induce the up-regulation of ERβ and promote cancer-stem-like properties [43]. These findings suggest the possibility that NNT-AS1 may have a similar ability to regulate ER receptors and contribute to tumor progression. These models are worthy of further exploration in future research.

## 3. The Diagnostic or Prognostic Value of Aberrant NNT-AS1 Expression

Some studies have analyzed the diagnostic and prognostic value of abnormal expression of NNT-AS1 (Table 2). The results of Farbod Esfandi et al. showed that the area under the curve (AUC) of NNT-AS1 expression level for gastric cancer diagnosis was 0.63, sensitivity was 73.3%, specificity was 70%, and *p* = 0.09, which suggests that NNT-AS1 is not suitable as a diagnostic marker for gastric cancer [34]. For breast cancer, the NNT-AS1 expression level showed lower sensitivity (56.6%) and similar specificity (71.2%) [37]. These two studies indicated that the diagnostic efficiency of the down-regulated NNT-AS1 may be unsatisfactory. In addition, the diagnostic value of up-regulated NNT-AS1 in cancer has not been reported.

Many studies have found that the expression level of NNT-AS1 is related to the prognosis of tumor patients. Studies on NSCLC [17], gastric cancer [32], HCC [21], cervical cancer [25,26], and bladder cancer [30] have shown that high-levels of NNT-AS1 were poor prognostic factors to reduce overall survival (OS). In addition, for CAA and osteosarcoma, up-regulated NNT-AS1 was reported to be significantly associated with reduced OS and disease-free survival (DFS) [22,24,27]. Through multivariate analysis, Li et al. found that a high level of NNT-AS1 is an independent prognostic factor leading to decreased OS in breast cancer patients [36]. The results of Wang et al. showed that a high level of NNT-AS1 can be used as an independent prognostic biomarker for OS and progression-free survival (PFS) in CRC patients [14]. These results indicate that NNT-AS1 may become a clinical biomarker for tumor prognosis.

## 4. NNT-AS1 Regulates Tumor Progression by Sponging miRNAs.

**Figure 2:**
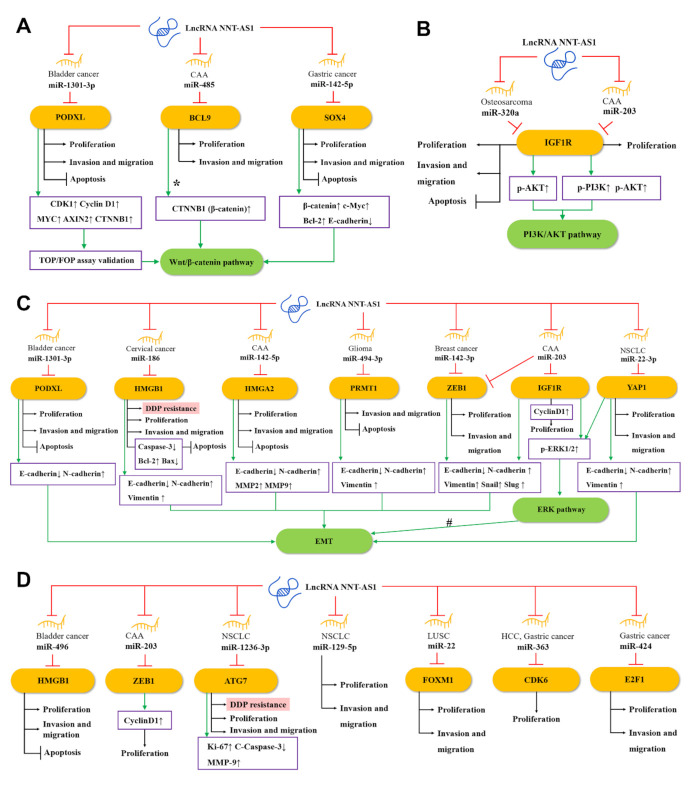
The competing endogenous RNA (ceRNA) mechanisms and potential downstream regulatory mechanisms of NNT-AS1. By sponging miRNAs, NNT-AS1 downregulates these miRNAs and influences downstream targets as well as (**A**) the Wnt/β–catenin pathway, (**B**) the PI3K/AKT signaling pathway, (**C**) the EMT and ERK signaling pathways, and (**D**) other ceRNA mechanisms, thereby affecting DDP resistance, proliferation, invasion, and metastasis, or inhibiting apoptosis of tumor cells. #: NNT-AS1/miR-22-3p is likely to regulate EMT through the YAP1-mediated ERK pathway. CCA, cholangiocarcinoma; NSCLC, non-small-cell lung cancer; LUSC, lung squamous cell carcinoma; HCC, hepatocellular carcinoma; EMT, epithelial–mesenchymal transition; DDP, cisplatin.

Salmena et al. proposed the competing endogenous RNA (ceRNA) hypothesis [44], which subsequently triggered a large number of studies to analyze complex ceRNA networks. The ceRNA hypothesis holds that transcripts with shared microRNA response elements (MREs) will compete with each other for post-transcriptional control. For example, lncRNA and mRNA (also pseudogenes, circular RNAs, or viral RNAs) with related MREs will compete to bind to miRNAs and be inhibited by them. Molecules such as lncRNA often have many different MREs and can sponge a large number of different miRNAs. The entire transcriptome will form a complex ceRNA network [44,45]. The ceRNA network has been recognized as a key regulator of cancer [46], and the NAT-AS1-centered ceRNA network can be representative. Through the NNT-AS1/miRNA/mRNA axis, NNT-AS1 can regulate the expression and protein activity of a series of downstream genes [44,47]. As shown in Figure 2, 15 miRNAs have been found to participate in the ceRNA network of NNT-AS1. At the same time, these miRNAs have at least 15 target mRNAs, which directly or indirectly participate in the biological behaviors of tumor cells such as epithelial–mesenchymal transition (EMT), proliferation, invasion, metastasis, apoptosis, and cisplatin resistance.

### 4.1. NNT-AS1 and the Wnt Signaling Pathway

As one of the most classic signaling pathways, the Wnt signaling pathway plays a key role in the occurrence, maintenance, and development of many cancers. Among them, the Wnt/β–catenin pathway mainly regulates the fate of cells during development [48]. As shown in Figure 2A, a study found that in bladder cancer, NNT-AS1 can up-regulate podocalyxin-like (PODXL) by sponging miR-1301-3p, which in turn activates the Wnt pathway-related proteins, including CDK1, cyclin D1, MYC, AXIN2, and CTNNB1 (β-catenin) [29]. In CAA, the up-regulated NNT-AS1 can compete with miR-485 to bind to B-cell CLL/lymphoma 9 protein (BCL9), thereby increasing the expression of BCL9 and promoting the proliferation, migration, and invasion of CCA cells [22]. BCL9 was found to be involved in the regulation of the Wnt/β–catenin pathway [49,50]. In addition, Zhang et al. found in GC cells that NNT-AS1 can regulate the expression of sex-determining region Y-related high-mobility-group-box transcription factor 4 (SOX4) through sponging miR-142-5p, while knockdown of NNT-AS1 will reduce β-catenin, c-Myc, and Bcl-2 but enhance e-cadherin expression. Knockdown of miR-142-5p and overexpression of SOX4 can reverse this performance, indicating that NNT-AS1 may activate the Wnt/β–catenin signaling pathway through the miR-142-5p/SOX4 axis, and then induce the proliferation, migration, invasion, and apoptosis inhibition of GC cells [35].

### 4.2. NNT-AS1 and the EMT and ERK Signaling Pathways

EMT is an evolutionarily-conserved developmental program. The dysregulation of EMT is related to tumorigenesis and can significantly enhance the ability of cancer cells to invade and metastasize [51]. As shown in Figure 2C, in CAA, NNT-AS1 can act as ceRNA to weaken the down-regulation of miR-142-5p on high mobility group AT-hook 2 (HMGA2), and lead to a significant down-regulation of e-cadherin, as well as a significant up-regulation of n-cadherin, MMP2, and MMP9. Therefore, the NNT-AS1/miR-142-5p/HMGA2 axis may promote the invasion and metastasis of CCA in part by enhancing EMT [24]. In another study of cervical cancer, bioinformatics analysis and experimental results showed that NNT-AS1 can upregulate high mobility group AT-hook 1 (HMGB1) by inhibiting miR-186 in vivo and in vitro. Knockdown of NNT-AS1 can promote caspase-3, e-cadherin, and bax, and inhibit the expression of bcl-2, n-cadherin, and vimentin. This indicates that the NNT-AS1/miR-496/miR-186/HMGB1 axis is related to EMT and cell apoptosis [26]. In addition, down-regulating NNT-AS1 in glioma cells can up-regulate e-cadherin and decrease the levels of n-cadherin and vimentin, which could be rescued by the miR494-3p inhibitor or protein arginine methyltransferase 1 (PRMT1) overexpression, suggesting that NNT-AS1 may increase the EMT of tumors through the miR-494-3p/PRMT1 axis [31].

ERK, an extracellular signal-regulated kinase of the mitogen-activated protein kinase (MAPK) pathway, has been proven to regulate a variety of cell behaviors, such as cell proliferation, invasion, migration, and stress [52]. In CCA cells, NNT-AS1 can competitively inhibit miR-203. Since zinc finger E-box binding homeobox 1 (ZEB1) is a potential target of miR-203, up-regulated NNT-AS1 can significantly increase the phosphorylation level of ERK1/2 and the level of EMT-related protein ZEB1 and reduce the expression of e-cadherin. The above effects can be reversed by miR-203 mimic, indicating the regulatory role of NNT-AS1/miR-203 in the ERK pathway and EMT [23]. In addition, NNT-AS1 can also increase Yes1-associated transcriptional regulator (YAP1) by sponging miR-22-3p, which in turn promotes the proliferation, migration, invasion, and EMT of NSCLC cells. Previously, YAP1 has been reported to be associated with EMT of various cancers such as NSCLC [53,54]. Therefore, NNT-AS1/miR-22-3p is likely to regulate EMT of NSCLC cells through the YAP1-mediated ERK pathway [17].

### 4.3. NNT-AS1 and PI3K/AKT Signaling Pathway

The PI3K/AKT pathway is often activated in a variety of human cancers and is considered a promising therapeutic target. Many positive regulators in this pathway have carcinogenic potential and have important effects on cell proliferation, survival, metabolism, invasion, and metastasis, as well as autophagy and aging [55]. As shown in Figure 2B, in osteosarcoma, NNT-AS1 can sponge miR-320a and up-regulate the expression of β-catenin, RUNX2, p-AKT, insulin-like growth factor type 1 receptor (IGF1R), MYC, cyclin D1, and MMP-13, thereby promoting osteosarcoma cell growth and tumor development [28]. Among them, β-catenin, RUNX2, and IGF1R have been proven to be direct targets of miR-320a [23,56,57], while IGF1R and p-AKT are able to activate the PI3K/AKT signaling pathway [58]. These provide evidence that NNT-AS1 is able to activate the PI3K/AKT signaling pathway through the ceRNA mechanism. Besides, IGF1R is a potential target of miR-203 [23]. In CAA cells, the NNT-AS1/miR-203/IGF1R axis has also been found to promote the phosphorylation of PI3K and AKT, which in turn activates the PI3K/AKT signaling pathway [23].

### 4.4. Other ceRNA Mechanisms of NNT-AS1

In bladder cancer, NNT-AS1 can serve as the ceRNA of miR-496 and increase the expression of the downstream gene HMGB1 [30]. In breast cancer, NNT-AS1 inhibits miR-142-3p competitively, thereby promoting the expression of ZEB1, which in turn induces the proliferation and metastasis of cancer cells [36]. In NSCLC, NNT-AS1 and miR-12363p competitively bind to autophagy-related gene 7 (ATG7), thereby affecting the proliferation, invasion, and metastasis of cancer cells [19]. NNT-AS1 can promote the proliferation and invasion of NSCLC cells by regulating miR-129-5p as well [16]. In another type of lung cancer LUSC, NNT-AS1 is a sponge of miR-22, and forkhead box M1 (FOXM1) is the direct target of miR-22. NNT-AS1 can directly regulate the expression of FOXM1 by inhibiting miR-22, thereby promoting the migration and invasion of LUSC cells, and inhibiting apoptosis [20]. In HHC, NNT-AS1 can sponge miR-363, reducing the targeted inhibition of cyclin-dependent kinase 6 (CDK6) by miR-363, thereby increasing the expression of CDK6 and promoting proliferation of cancer cells [21]. In GC, NNT-AS1 sponges miR-424 to up-regulate E2F transcription factor 1 (E2F1), thus promoting the cell cycle progression of GC cells and inhibiting apoptosis [32] (Figure 2D).

## 5. NNT-AS1 and Cisplatin Resistance

As a platinum-based drug, cisplatin can be widely used in the treatment of various malignant solid tumors, such as lung cancer, ovarian cancer, head and neck cancer, and CRC. [59]. Despite the initial response rate being relatively consistent, cisplatin is prone to drug resistance and subsequent chemotherapy failure. Therefore, many studies have explored the molecular mechanism of overcoming cisplatin resistance [60]. Recently, there is evidence that lncRNA is involved in regulating drug delivery and drug resistance [51], some of which can mediate cisplatin resistance in cancer, such as lncRNA XIST [61], lncRNA UCA1 [62], and lncRNA CCAT1 [63]. Recent research evidence shows that NNT-AS1 is also related to cisplatin resistance. Cai et al. first found that NNT-AS1 was significantly up-regulated in drug-resistant tissues and cells of NSCLC, and when down-regulating NNT-AS1, the levels of p-MAPK1 and Slug in cisplatin-resistant cancer cells were significantly reduced, suggesting that NNT-AS1 may induce cisplatin resistance through the MAPK/Slug signaling pathway in NSCLC [18]. Wang et al. also confirmed the correlation between NNT-AS1 and cisplatin resistance in NSCLC cells and mouse models, and further revealed that NNT-AS1 may mediate cisplatin resistance through the miR-1236-3p/ATG7 axis [19] (Figure 2D). In addition, Liu et al. found abnormal up-regulation of NNT-AS1 in cisplatin-resistant cervical cancer tissues and cells, and loss-of-function assays confirmed the relationship between NNT-AS1 and cisplatin resistance. Further restoration experiments showed that NNT-AS1 knockdown can antagonize cisplatin resistance by adjusting the miR-186/HMGB1 axis [26] (Figure 2B).

Given the central position of NNT-AS1 in the ceRNA network, NNT-AS1 targeting has the potential to broaden the landscape of therapeutic interventions. However, compared with mRNA and miRNA, technology of targeting lncRNA in vivo is still in its infancy. Taking RNA interference (RNAi) technology as an example, animal models have confirmed that siRNA-mediated lncRNA knockdown can reduce the proliferation, growth, and the metastasis of cancer cells [64,65]. Since NNT-AS1 is mainly located in the cytoplasm, current studies mostly use short hairpin RNA (sh-RNA) to achieve RNAi. In addition, the feasibility of antisense oligos (ASOs) targeting lncRNA in vivo has also been confirmed in different mice models of cancer [66,67]. However a few studies have shown that ASO-based therapeutic drugs tend to accumulate in the liver and kidney [68,69]. Therefore, how to effectively deliver drugs to target tissues or target cells is one of the main challenges faced by this method. In recent years, clustered regulatory interspaced short palindromic repeats-associated endonuclease 9 (CRISPR/Cas9) genome editing technology and its derived CRISPR interference/activation (CRISPRi/a) and CasRx technology [70] have provided a more specific means for regulating lncRNA. For NNT-AS1, its genomic region contains a large number of screenable CRISPR targets and also encodes many regulatory elements (Figure 1), so this technology may bring unnecessary off-target effects. In fact, mainstream technologies currently used to regulate lncRNA have more or less off-target effects [71]. However, with the deepening of our understanding of lncRNA and the advancement of gene-editing technology, we look forward to NNT-AS1 being more effectively regulated and used to solve more clinical problems in the future.

## 6. Conclusions

In general, lncRNA NNT-AS1 is up-regulated in most tumor types and plays an important role in the progression of malignant tumors through the regulation of complex ceRNA networks. NNT-AS1 may activate the Wnt signaling pathway, the ERK signaling pathway, the PI3K/ AKT signaling pathway, and the MAPK/Slug signaling pathway, etc. Many studies have shown that NNT-AS1 dysregulation is related to tumor prognosis, especially in breast cancer and CRC, in which the abnormal expression level of NNT-AS1 is an independent prognostic marker. Recent studies also revealed the involvement of NNT-AS1 in mediating cisplatin resistance and cell experiments and mouse models have confirmed that down-regulating NNT-AS1 can enhance the sensitivity to cisplatin, which provides a new idea for overcoming cisplatin resistance.

It is worth noting that NNT-AS1 was down-regulated in studies [34,37,38,39] on ovarian cancer, triple-negative breast cancer, and gastric cancer, which is inconsistent with the results of the others [32,33,35,36]. These differences need to be further verified in large-scale experiments, and the connection between NNT-AS1 and sex hormones and sex hormone receptors deserves more in-depth exploration. Moreover, the diagnostic value of NNT-AS1 in cancer remains to be confirmed.

In the future, we believe that more research will focus on NNT-AS1 and its targets and make a more detailed analysis of the molecular mechanism of NNT-AS1. This will lay a solid theoretical foundation for its application in clinical-targeted therapy, predicting tumor prognosis and chemotherapy resistance.

## Figures and Tables

**Table 1:** The role of NNT-AS1 in different human cancers.

Tumor Type	Expression Level	Number of Clinical Samples	Assessed Cell Lines	Effect In Vitro	Effect In Vivo	Regulatory Mechanism	Ref.
NSCLC	Up-regulated	1037 tumor samples and 108 normal samples from TCGA database; 43 paired tissues	H1299, H23, A549, SPC-A-1, H522	Proliferation ↑, migration and invasion ↑	--	NNT-AS1/miR-129-5p axis	[16]
	Up-regulated	37 paired tissues	H1650, PC-9, A549, H1299	Proliferation ↑, migration and invasion ↑, EMT ↑	Tumor growth ↑	NNT-AS1/miR-22-3p/YAP1 axis	[17]
	Up-regulated	10 paired tissues of DDP resistant and non-resistant	A549 and SPCA-1 of DDP resistant and non-resistant	DDP resistance ↑, proliferation ↑, colony formation ↑, cell cycle arrest ↓, apoptosis ↓	--	Activate MAPK/Slug signaling pathway	[18]
	Up-regulated	31 paired tissues	H1299, H23, H522, A549 of DDP resistant and non-resistant	DDP resistance ↑, proliferation ↑, poptosis ↓, migration and invasion ↑	--	NNT-AS1/miR-1236-3p/ATG7 axis	[19]
LUSC	Up-regulated	46 paired tissues		Apoptosis ↓, migration and invasion ↑	--	NNT-AS1/miR-22/FOXM1 axis	[20]
CRC	Up-regulated	70 paired tissues	SW480, SW620	Proliferation ↑, migration and invasion, colony formation ↑, cell cycle arrest ↑, EMT ↑	Tumor growth ↑ Liver metastasis ↑	Activate MAPK/Erk signaling pathway	[14]
Gastric cancer	Up-regulated	48 paired tissues	BGC-823, MGC-803, AGS, SGC-7901, MKN-45	Proliferation ↑, cell cycle arrest ↓, migration and invasion ↑	Tumor growth ↑	NNT-AS1/miR-424/E2F1 axis	[32]
	Up-regulated	40 paired tissues	HGC-27, MGC-803, MKN-45, SGC-7901	Proliferation ↑, cell cycle arrest ↓, migration and invasion ↑	--	NNT-AS1/miR-363 axis	[33]
	Down-regulated	30 paired tissues	--	--	--	--	[34]
	Up-regulated	25 paired tissues	AGS, HGc-27	Proliferation ↑, apoptosis ↓, migration and invasion ↑	--	miR-142-5p/SOX4/Wnt/β-catenin signaling pathway	[35]
HCC	Up-regulated	42 paired tissues	HepG2, Huh7	Proliferation ↑, colony formation ↑, apoptosis ↓, cell cycle arrest ↓	Tumor growth ↑	NNT-AS1/miR-363/CDK6 axis	[21]
Cholangiocarci-noma	Up-regulated	48 paired tissues	Huh28, KMBC, HCCC9810, HuCCT1, RBE, CCLP1, QBC-939	Proliferation ↑, colony formation ↑, migration and invasion ↑	Tumor growth ↑	NNT-AS1/miR-485/BCL9	[22]
	Up-regulated	20 paired tissues	SG231, CCLP1, HuCCT1, TFK1	Proliferation ↑, EMT ↑	--	NNT-AS1/miR-203/IGF1R/ZEB1; activate PI3K/AKT and ERK1/2 pathways	[23]
	Up-regulated	Cohorts from TCGA database (number not shown); 30 paired tissues	RBE, HuCCT1, QBC939, TFK1	Proliferation ↑, migration and invasion ↑, EMT ↑	Tumor growth ↑	NNT-AS1/miR-142-5p/HMGA2 axis	[24]
Cervical cancer	Up-regulated	79 paired tissues	SiHa, CaSki	Proliferation ↑, migration and invasion ↑, cell cycle arrest ↑, apoptosis ↓	Tumor growth ↑	Activate Wnt/β–catenin pathway	[25]
	Up-regulated	24 (chemo-sensitive) + 34 (chemo-resistant) paired tissues	HeLa, SiHa	DDP resistance ↑, proliferation ↑, Migration and invasion ↑, EMT ↑, apoptosis ↓	DDP resistance ↑Tumor growth ↑	NNT-AS1/miR-186/HMGB1 axis	[26]
Breast cancer	Up-regulated	64 paired tissues	MDA-MB-468, MCF-7, MD-MB-231	Proliferation ↑, colony formation ↑, migration and invasion ↑, EMT ↑	--	NNT-AS1/miR-142-3p/ZEB1 axis	[36]
	Down-regulated	54 paired tissues	--	--	--	--	[37]
	Down-regulated *	237 tumor samples and 11 normal samples from GEO datasets	--	--	--	--	[38]
Ovarian cancer	Down-regulated	55 paired tissues	HO-8910PM, HO-8910, OVCAR3, SK-OV-3	Colony formation ↓, cell cycle arrest ↑, migration and invasion ↓, apoptosis ↑	--	--	[39]
Osteosarcoma	Up-regulated	126 paired tissues and 18 healthy controls	U2OS, Saos2, OS-9901, SOSP-9607, MG-63, OS-732	Proliferation ↑, colony formation ↑, cell cycle arrest ↓, apoptosis ↓, migration and invasion ↑	Tumor growth ↑	AKT, C-myc, CyclinD1, BCL-2, MMP-2, MMP-9	[27]
	Up-regulated	--	OS-732, U2OS	Proliferation ↑, colony formation ↑, apoptosis, migration and invasion ↑	Tumor growth ↑	NNT-AS1/miR-320a; Activate PI3K/AKT signaling pathway	[28]
Bladder cancer	Up-regulated	--	RT-4, UM-UC-3, J82, T24	Proliferation ↑, colony formation ↑, migration and invasion ↑, EMT ↑, apoptosis ↓	--	NNT-AS1/miR-1301-3p/PODXL axis; activate Wnt pathway	[29]
	Up-regulated	47 paired tissues	T24, 5637, UM-UC-3, TCC-SUP	Proliferation ↑, migration and invasion, apoptosis ↓	--	NNT-AS1/miR-496/HMGB1 axis	[30]
Glioma	Up-regulated	73 paired tissues	U87, LN229, U251	Proliferation, cell cycle arrest ↓, migration and invasion ↑, EMT ↑, apoptosis ↓	--	NNT-AS1/miR-494-3p/PRMT1 axis	[31]

* Triple-negative breast cancer; NSCLC, non-small-cell lung cancer; LUSC, lung squamous cell carcinoma; CRC, colorectal cancer; HCC, hepatocellular carcinoma; TCGA, The Cancer Genome Atlas; GEO, Gene Expression Omnibus; EMT, epithelial–mesenchymal transition; DDP, cisplatin; ↑, promotion; ↓, inhibition.

**Table 2:** Diagnostic or prognostic value of NNT-AS1 expression.

Cancer Type	Sample Size	Expression Pattern	Diagnostic/Prognostic Value	Ref.
NSCLC	37 patients	Up-regulated	Prognostic factor of OS	[17]
Gastric cancer	30 patients	Down-regulated	AUC = 0.63, sensitivity = 73.3%, specificity = 70%	[34]
	48 patients	Up-regulated	Prognostic factor of OS	[32]
CRC	70 patients	Up-regulated	Independent prognostic factor of OS and PFS	[14]
HCC	42 patients	Up-regulated	Prognostic factor of OS	[21]
Cholangiocarcinoma	48 patients	Up-regulated	Prognostic factor of OS	[22]
	30 patients	Up-regulated	Prognostic factor of OS and DFS	[24]
Cervical cancer	79 patients	Up-regulated	Prognostic factor of OS	[25]
	58 patients	Up-regulated	Prognostic factor of OS	[26]
Breast cancer	64 patients	Up-regulated	Independent prognostic factor of OS	[36]
	54 patients	Down-regulated	AUC not shown, specificity = 71.2%, sensitivity = 56.6%	[37]
Bladder cancer	47 patients	Up-regulated	Prognostic factor of OS	[30]
Osteosarcoma	126 patients	Up-regulated	Prognostic factor of OS and DFS	[27]

NSCLC, non-small-cell lung cancer; CRC, colorectal cancer; HCC, hepatocellular carcinoma; OS, overall survival; AUC, area under the curve; PFS, progression-free survival; DFS, disease free survival.
